# Non-Surgical Retreatment of Maxillary Lateral Incisor with Unusual Anatomy: A Case Report and Mini Review

**DOI:** 10.22037/iej.v12i3.16607

**Published:** 2017

**Authors:** Ashraf Shubbar, Behnam Bolhari, Nooshin Fakhari, Parvin Alemi, Ali Nosrat

**Affiliations:** a * Department of Endodontics, Dental School, Tehran University of Medical Sciences, Tehran, Iran;*; b *Iranian Center for Endodontic Research, Research Institute of Dental Sciences, Dental School, Shahid Beheshti University of Medical Sciences, Tehran, Iran;*; c *Department of Endodontics, Periodontics, and Prosthodontics, Dental School, University of Maryland Baltimore, Baltimore, Maryland, USA*

**Keywords:** Maxillary Lateral Incisor, Retreatment, Root Canal Anatomy

## Abstract

Knowledge about internal anatomy plays a crucial role in the success of the root canal treatment. Many studies on internal anatomy have repeatedly reported that maxillary lateral incisors have only one canal. The primary aim of this article was to describe successful non-surgical retreatment of a permanent maxillary lateral incisor with two root canals and open apices. The treatment was carried out using dental operating microscope and the canals were obturated with mineral trioxide aggregate (MTA) as an apical plug. A review of literature was also conducted to evaluate the anatomical variations of maxillary lateral incisors.

## Introduction

Adetailed knowledge of root canal anatomy is necessary to effectively perform endodontic treatment [1, 2]. Maxillary lateral incisor is identified as a single-rooted tooth with a single canal (one orifice with one apical foramen) in several studies [3, 4]. However there are few reports showing anatomical variations including two root canals [5, 6], two roots [7-13], three root canals [14] and even four root canals [15]. Most of these studies have identified these findings as uncommon variations associated with tooth anomalies including dens invaginatus, talon cusp, peg-shaped lateral incisor, gemination and fusion [16]. The primary aim of this article was to present a case of non-surgical retreatment of a maxillary lateral incisor with two root canals without any developmental anomalies. The secondary aim was to conduct a review on all reported cases of maxillary lateral incisors with more than one canal.

## Case Report

A 14 year-old male was referred to the Endodontic Department of Tehran Dental School, Tehran University of Medical Sciences with the chief complaint of pain and tenderness on palpation of the right maxillary lateral incisor. Patient’s medical history was non-contributory.

**Figure 1.**
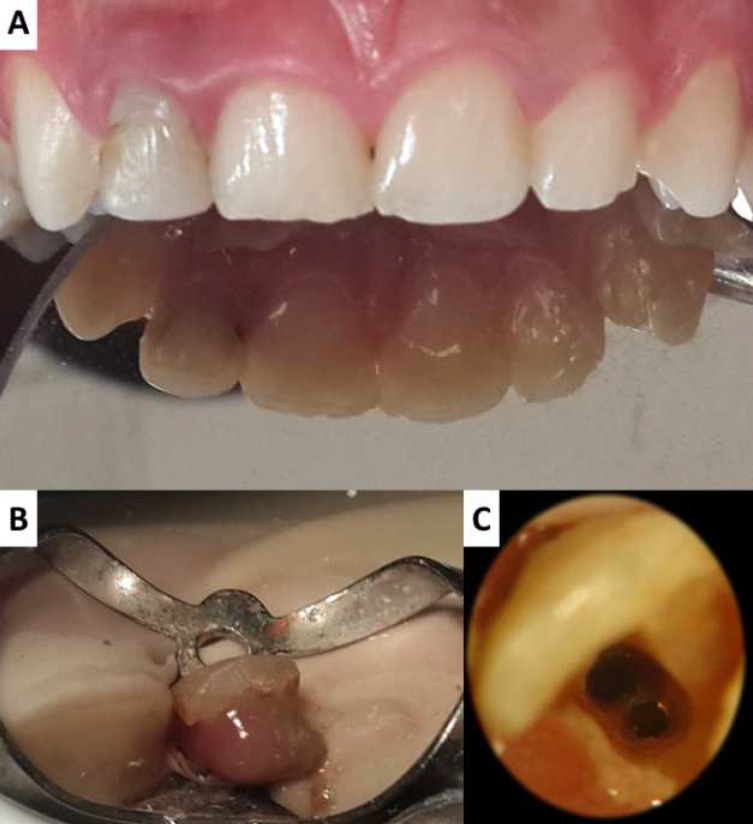
A) Clinical view of the anterior teeth, showing cervical discoloration, palatal composite filling and mesial caries on tooth #12; B) Pus discharge through the access cavity in #12 after removal of the previous root canal filling material; C) View of the access cavity through microscope. Two canals, labial and palatal, are visible

Upon clinical examinations, tooth #12 was sensitive to percussion and palpation. The probing depths and mobility were within normal limits (<3 mm and <1 mm labially and palatally, respectively). No soft tissue swelling was noted. Cold test and electric pulp test (DTS, Guangdong, China Mainland) were done on anterior teeth that showed all tested teeth being normal except tooth #12 which responded negative to both tests. Tooth #12 was normal in shape with no clinically visible anomalies in the crown. A slight cervical discoloration as well as a carious lesion on the mesial side was noted. There was a palatal composite restoration with open margins (Figure 1A).

**Figure 2:**
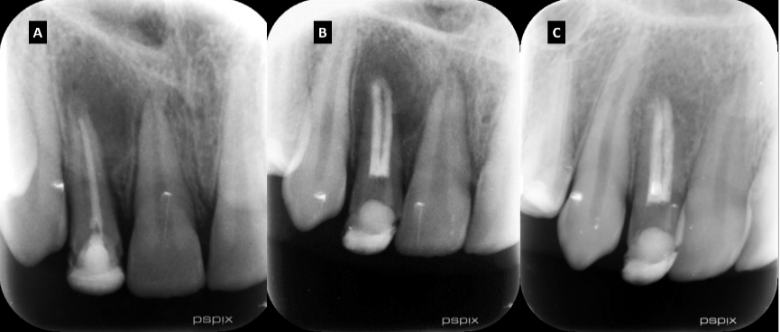
A) Pre-operative radiographic view of tooth #12. Note the large periapical lesion extended coronally on the mesial side of the root, the immature apex of the tooth, and the inadequate root canal treatment; B) After retreatment and coronal restoration. The apical third of both canals obturated with MTA and the rest of the root canal spaces obturated with warm vertical compaction of gutta-percha; C) 18-months follow up tooth #12. Please not the complete osseous healing of the periapical lesion

Radiographic examination revealed inadequate previous root canal treatment in tooth #12, associated with a periapical and lateral radiolucent lesion. Tooth #12 was immature (Figure 2A). There was no radiographic evidence of any tooth abnormalities or extra roots. The initial radiography was taken with a slight mesial shift. The previous root canal filling material was deviated mesially suggesting the possibility of a missed canal on the labial side (Figure 2A). The diagnosis was symptomatic apical periodontitis. The recommended treatment was non-surgical retreatment of tooth #12. 

Under local anesthesia (buccal infiltration of 1.8 mL Lidocaine 2% with epinephrine 1:80000; DarouPakhsh, Tehran, Iran) and rubber dam isolation, an access cavity was prepared using high-speed handpiece and a round carbide bur (E 0123, Dentsply Maillefer, Ballaigues, Switzerland). The gutta-percha in the coronal and middle third was removed with Gates Glidden drills #3 and 2 (Lexicon Gates Glidden, Dentsply, Tulsa Dental, Tulsa, OK, USA), respectively. The gutta-percha in the apical third was first softened with chloroform solvent (Chloroform, Sultan Chemists, Englewood, NJ, Inc. USA), then removed with size #30 Hedstrom file (Dentsply Maillefer, Ballaigues, Switzerland). Pus discharge was observed immediately after removal of the gutta-percha (Figure 1B). After careful examination under magnification using a dental operating microscope (OPMI pico, Carl Zeiss Meditec, Inc, Dublin, CA, USA), a second canal was located on the labial side (Figure 1C).

Cleaning and shaping of the canals were performed with hand instrumentation (passive step-back technique) and the root canal spaces were irrigated with 15 mL of 3% sodium hypochlorite (Vishal Dentocare Ltd, Ahmedabad, India) intermittently. Since both apices were open the master apical file was #90 for both canals. A creamy paste of calcium hydroxide (Henry Schein Company, Melville, NY, USA) was prepared and placed into the canals using lentulo spiral [17]. The access cavity was sealed with zinc oxide eugenol based temporary filling material zonalin (Kemedent, UK).

The patient was recalled one week later. The symptoms were resolved. After local anesthesia and rubber dam isolation, calcium hydroxide was removed from the canals by irrigation with 10 mL of 3% sodium hypochlorite and H-file #60. Ortho MTA powder (BioMTA, Seoul, Republic of Korea) was mixed with saline to prepare a putty consistency and was packed into both canals to the working length as an apical plug. Saline wetted paper points were placed over the MTA inside the canals and the access cavity was sealed with Zonalin. At the third appointment, two days later, the MTA setting was checked under rubber dam isolation and the rest of the root canal spaces were obturated with gutta-percha (Aryadent, Tehran, Iran) and AH-26 silver free sealer (Dentsply, DeTrey, Konstanz, Germany) using warm vertical compaction technique. The access cavity was sealed with Zonalin and the patient was referred to the Operative Dentistry Department for coronal restoration (Figure 2B).

The patient was recalled 3, 6, 12 and 18 months after treatment. Tooth #12 was functional and asymptomatic. Probing depths and mobility were within normal limits. There was no sensitivity to percussion or palpation. The radiographic examination showed complete osseous healing (Figure 2C).

## Discussion

A Medline database search *via* PubMed search engine (www.ani.nlm.nih.gov/pubmed) and a search on the Web of Science (Thomson Reuters) (www.Weboflmowledge.com) were done on the anatomy of maxillary lateral incisors using the following keywords: ”Multiple” OR ”accessory” OR ”double” OR ”extra” OR ”two” OR ”three” OR ”four” OR ”five” OR ”Six” AND ”Maxillary lateral incisors” AND ”Root” OR ”Canal”. All the reports on maxillary lateral incisors with more than one roots and/or canals are included in the review. The reports on gemination or fusion with a supernumerary tooth were excluded. The following data were extracted: Year of publication, number of cases reported, patient age, number of roots, number of canals, location of the canals, the associated developmental anomaly, and the type of treatment. The data are presented in Table 1.

Our review of literature (Table 1) showed that most cases of maxillary lateral incisors with abnormal canal configuration were reported to have morphological or developmental anomaly, (*e.g.* radicular groove, dens invaginatus, peg shaped tooth, talon cusp, *etc.*). Overall, maxillary lateral incisors with dens invaginatus usually have complex root canal systems [18]. The presented case, however, did not have any morphological anomalies.

**Table 1:** Previous case reports of maxillary lateral incisor with multiple roots or canals (MD: mesiodistally; BP: Buccopalatally; RCT: root canal treatment; 1-2: one canal branched to two; Tx: treatment; ND: not defined

**Article**	**Year**	**N **	**Gender**	**Age**	**Roots**	**Canals**	**A**	**Anomaly**	**Tx**
**Christie ** ***et al.*** ** [27]**	1981	2	F F	35 21	2 2	2 2	MD MD	ND Extra cusp	RCT RCT+Root-end surgery
**Zillich ** ***et al.*** ** [28]**	1983	1	M	31	2	2	ND	Dens in dente	RCT
**Fried & Winter [29]**	1984	1	F	28	2	2	BP	Palatal groove	RCT
**Thompson ** ***et al.*** ** [30]**	1985	1	M	37	1	2	MD	ND	RCT
**Vire De [31]**	1985	1	F	27	2	2	BP	ND	Extracted
**Greenfeld ** ***et al.*** ** [32]**	1986	4	M M M M	9 23 42 41	2 2 2 2	2 2 2 2	BP BP BP BP	Dens in dente ND Radicular groove Radicular groove	RCT+root amputation RCT+root amputation RCT+perio management RCT+perio management
**Yoshikawa ** ***et al. *** **[13]**	1987	1	F	13	2	2	1-2	Palatal groove	RCT
**Hatton & Ferrillo [5]**	1989	1	M	26	2	2	MD	ND	Root-end surgery
**Campos [33]**	1990	1	M	24	2	2	BP	Palatal grooveTalon cusp	RCT
**Pecora & Santana [19]**	1991	1	M	40	2	2	ND	ND	RCT
**Platt[34]**	1995	1	F	48	2	2	ND	ND	Extracted
**Walvekar & Behbehani [14]**	1997	1	F	19	2	3	MD	Dens in Dente	RCT
**Peix‐Sánchez M. [35]**	1999	1	F	19	1	3	MD P	Larger crown	RCT
**Collins [7]**	2001	1	M	28	2	2	MD	ND	Incomplete treatment
**Low & Chan [16]**	2004	2	F/M	27 23	1 2	2 2	MD BP	ND Palatal groove	RCT RCT
**M. Jung [36]**	2004	1	M	12	1	3	MD P	Dens in dente	RCT
**Shokouhinejad ** ***et al.*** ** [6]**	2009	1	F	12	2	2	BP	ND	RCT
**Venugopal and Srirekha [37]**	2010	1	M	24	2	2	MD	ND	RCT
**Altuntas ** ***et al.*** ** [38]**	2010	1	F	10	1	2	MD	Dens in dente	RCT
**Ghoddusi ** ***et al.*** ** [39]**	2010	1	M	26	1	2	BP	ND	RCT
**Gandhi ** ***et al.*** ** [8]**	2011	1	M	30	2	2	BP	Radicular groove	Root resection
**Ravindranath et al. [11]**	2011	1	F	27	2	2	MD	ND	RCT
**Dexton ** ***et al.*** ** [40]**	2011	1	M	25	2	2	MD	Normal	RCT
**Mohan ** ***et al.*** ** [10]**	2012	1	F	25	2	2	MD	ND	RCT
**Matta[12]**	2012	1	M	20	2	2	MD	ND	RCT
**Helvacioglu& Aydemir [41]**	2012	1	M	16	1	2	MD	Dens in dente	RCT
**Makade ** ***et al.*** ** [42]**	2013	1	M	27	2	2	ND	ND	RCT
**Subbiya ** ***et al.*** ** [43]**	2013	1	M	16	1	2	MD	Dens in dente	RCT
**Jaikailash ** ***et al.*** ** [44]**	2014	1	M	17	5	1	ND	Peg shape, Dens in dente	RCT
**Hoseini & Abbaszadegan [9]**	2014	1	F	16	2	2	BP	ND	RCT
**Nosrat& Schneider [15]**	2015	1	M	16	2	4	MD-BP	Dens in dente	RCT
**Aydemir ** ***et al.*** ** [45]**	2015	1	F	65	2	2	BP	Normal	RCT
**Çalışkan ** ***et al.*** ** [46]**	2016	1	M	16	2	2	BP	Dens in dente	surgical amputation

Maxillary lateral incisors are located at a site of high embryological risk [19]. This tooth occasionally shows a reduction in size [20], but the size can be similar to central incisor. It frequently shows different crown shapes, for example, peg-shaped, cone-shaped, barrel-shaped and canine-shaped. 

The reasons why some lateral incisors have two roots or two canals is still unknown, but there are some presumptions explaining the reason(s) for supernumerary/extra root(s) in anterior teeth. Kelly [21] presumed that traumatic injuries to the surface of the root or to the forming periodontium (Hertwig’s epithelial root sheath) at the time of root formation can be a cause. The term *supernumerary root* was used by Neville [22] to describe presence of an unusual extra root.

The presented report demonstrates the importance of accurate radiographic analysis before treatment interventions. CBCT imaging gives the clinician the opportunity of visualizing the internal and external anatomy of the tooth in three dimensions. Therefore, it increases the probability of finding canals and avoiding procedural errors [15, 23]. On the other hand, it is worth noting that CBCT has drawbacks and short comings. Scattered radiation creates noise around radio-opaque materials which makes it difficult to explore the internal anatomy in retreatment cases [24]. Retrospective evaluation of the presented case proves that three dimensional imaging could have been a helpful diagnostic tool to explore the internal anatomy of the tooth.

It is now well-documented that using magnification enhances the clinician’s ability to visualize the anatomy of the pulp chamber and canals [25]. Studies have shown that dental operating microscope is superior to magnifying loups in locating and negotiating the canals [26]. The use of dental operating microscope played a crucial role in exploring the internal anatomy of the presented case.

## Conclusion

The present report highlights the possibility of unusual root canal anatomy in maxillary lateral incisors even in the absence of tooth anomalies. Clinicians should be aware of the variations and be able to recognize and clinically managing these variants; these rare cases may demands a specialist’s skill and knowledge. 
